# Rapid DNA barcoding analysis of large datasets using the composition vector method

**DOI:** 10.1186/1471-2105-10-S14-S8

**Published:** 2009-11-10

**Authors:** Ka Hou Chu, Minli Xu, Chi Pang Li

**Affiliations:** 1 Department of Biology, The Chinese University of Hong Kong, Hong Kong, PR China; 2 Molecular Biotechnology Programme, The Chinese University of Hong Kong, Hong Kong, PR China; 3 Department of Bioinformatics and Genomics, University of North Carolina at Charlotte, NC28223, USA

## Abstract

**Background:**

Sequence alignment is the rate-limiting step in constructing profile trees for DNA barcoding purposes. We recently demonstrated the feasibility of using unaligned rRNA sequences as barcodes based on a composition vector (CV) approach without sequence alignment (*Bioinformatics *22:1690). Here, we further explored the grouping effectiveness of the CV method in large DNA barcode datasets (COI, 18S and 16S rRNA) from a variety of organisms, including birds, fishes, nematodes and crustaceans.

**Results:**

Our results indicate that the grouping of taxa at the genus/species levels based on the CV/NJ approach is invariably consistent with the trees generated by traditional approaches, although in some cases the clustering among higher groups might differ. Furthermore, the CV method is always much faster than the K2P method routinely used in constructing profile trees for DNA barcoding. For instance, the alignment of 754 COI sequences (average length 649 bp) from fishes took more than ten hours to complete, while the whole tree construction process using the CV/NJ method required no more than five minutes on the same computer.

**Conclusion:**

The CV method performs well in grouping effectiveness of DNA barcode sequences, as compared to K2P analysis of aligned sequences. It was also able to reduce the time required for analysis by over 15-fold, making it a far superior method for analyzing large datasets. We conclude that the CV method is a fast and reliable method for analyzing large datasets for DNA barcoding purposes.

## Background

In 2003, Hebert et al [1] proposed to use a 648-bp region from the 5'-end of the cytochrome *c *oxidase subunit 1 (COI) gene as a DNA barcode for identifying all metazoan species. The final goal of DNA barcoding is to identify all eukaryotic species [2,3]. Recently, DNA barcoding has been tested on several groups of organisms including birds [4], fishes [5] and amphibians [6] with promising results. Ratnasingham and Hebert [7] estimated that the barcode of life data system (BOLD) would eventually generate 100 million records (for COI-barcoding only) twice the current size of the GenBank database, and that enterprise-scale software would be needed to handle such a large dataset. The goal of BOLD is to generate a "taxon ID tree" based on a neighbour-joining (NJ) algorithm for every query sequence for easy identification. As in other traditional methods of tree construction, sequence alignment has to be performed before construction of the K2P/NJ-tree (i.e., a NJ tree based on the Kimura-2-parameter, K2P, distance). In BOLD, alignment is executed based on the hidden Markov models [7]. Due to the high computational burden involved, BOLD has unfortunately been unable to incorporate all data records in constructing the K2P/NJ tree. The short-term solution is to divide the large barcode dataset into several "sub-projects" with a size limit of 5,000 specimens each for analysis [7]. However, as an estimated 200,000 additional barcode records will be entered in the database each year [8], the limit of 5,000 specimens for each sub-project will be quickly saturated because closely related taxa (sequences) should not be divided into subsets but preferably analyzed together. The long-term solution is therefore to develop more efficient analytical methods as alternatives or supplements for handling such a large dataset.

COI has several claims to be a suitable DNA barcode marker, including ease in amplification across a wide variety of organisms and provision of enough information to enable organisms to be identified to the species level. But it also has its drawbacks, including inherent risks due to maternal inheritance (noticeably failure in detecting hybridization), the presence of pseudogenes (numts), and its inconsistent evolutionary rate among lineages [2,9,10]. These disadvantages continue to disappoint biologists hoping to rely on single gene as the sole marker for taxonomic identification [6,11,12]. The feasibility of using alternative and additional genes as DNA barcodes has been explored [9,13,14]. Ribosomal RNA (rRNA) genes and their internal transcribed spacer (ITS) [6,15] show promise as DNA barcode markers for distinguishing different organisms and, as COI is highly conserved in plants, barcode markers such as the *trnH-psbA *spacer and *rbcL *gene [9,11,16] have also been proposed. Yet because of the high frequency of insertions/deletions in the rRNA and the other non-protein-coding markers, sequence alignment is a critical step in the analysis. This, in turn, requires a large amount of additional computational power during the alignment step, further pushing the analytical power of BOLD system toward its limit. The process of sequence alignment is not only very costly in computational power, but has also yet to be standardised. A lack of standard protocols and inconsistencies between the aligned datasets of different laboratories represent a drawback [13,17-21] to the incorporation of any non-protein-barcoding markers in BOLD. Including markers such as rRNA in BOLD would mean that alignment will be executed automatically and any problems in alignment cannot be adjusted manually. The problems associated with the alignment procedure effectively limit the use of DNA barcoding [13].

Our previous study [13] demonstrated that the composition vector (CV) method [22] was an effective and efficient tree construction method for analyzing rRNA datasets, thereby facilitating the use of these genes as DNA barcodes. We believe that the CV approach can also sidestep the problems associated with sequence alignment in analyzing large datasets of both COI and non-protein-coding barcode markers, the latter of which usually involve sequence length variation even among closely related taxa. In the present study, we analyzed large DNA barcode datasets, each with more than 300 sequences (including non-COI datasets with variable sequence lengths) with the CV method. Sequences from three published DNA barcode datasets available from GenBank, namely birds [23], fishes [5] and nematodes [14], were analyzed. A 16S rRNA dataset of decapod crustaceans containing 466 GenBank sequences and 268 sequences generated in our laboratory was also analyzed. These datasets were chosen because they included the most common genetic markers that have been proposed as DNA barcodes, including COI, 16S rRNA and nuclear SSU rRNA genes, and contained the largest number of DNA sequences, ranging from 349 to 754, so far assembled. The aim of our study was to evaluate how well and how fast the CV method could handle these large barcode datasets without sequence alignment.

## Results

### Bird dataset

**Figure 1:**
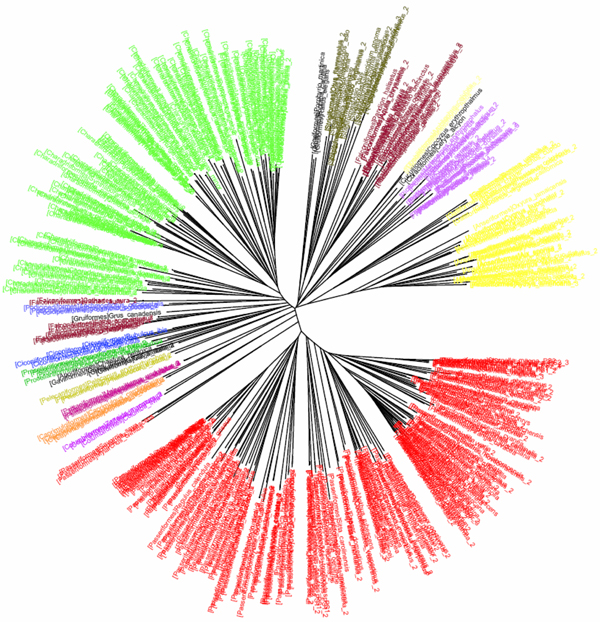
**K2P/NJ tree based on COI dataset of North American birds (Herbert et al 2004).**Sequences are labelled in different colours according to the orders of that taxa.

**Figure 2:**
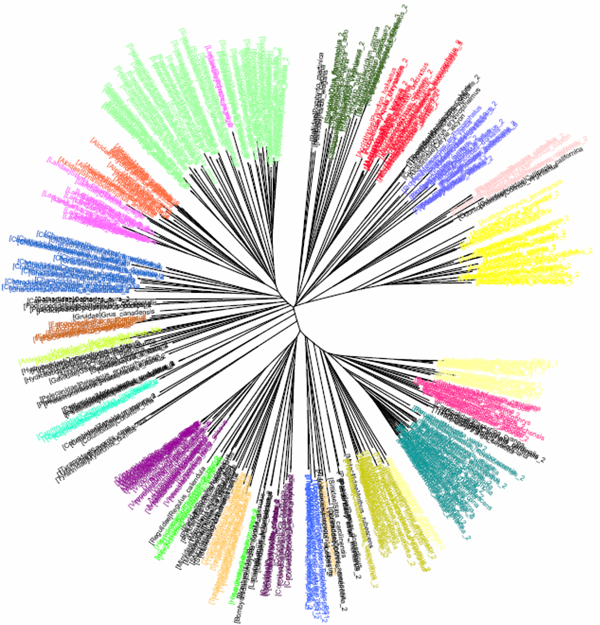
**CV tree (*K*=9) based on COI dataset of North American birds (Herbert et al 2004).**Sequences are labelled in different colours according to the orders of the taxa.

**Figure 3:**
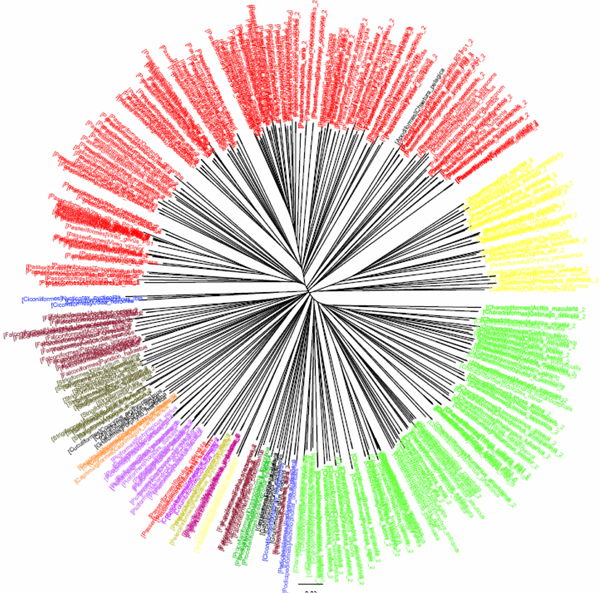
**K2P/NJ tree based on COI dataset of North American birds (Herbert et al 2004).**Sequences are labelled in different colours according to the families of the taxa

**Figure 4:**
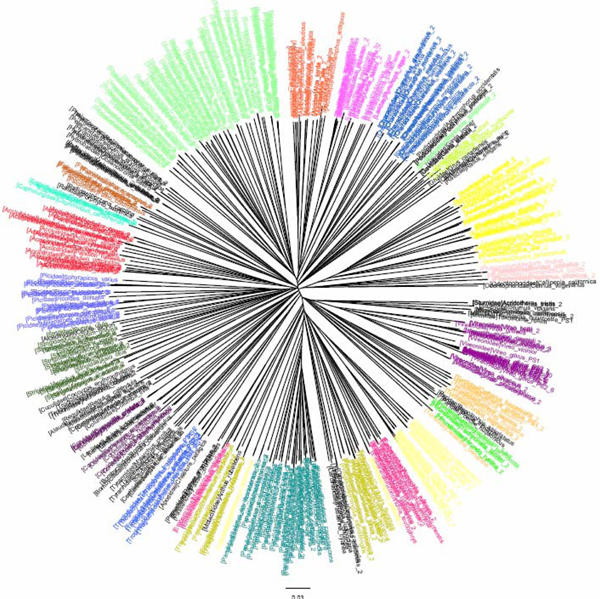
**CV tree (*K*=9) based on COI dataset of North American birds (Herbert et al 2004).**Sequences are labelled in different colours according to the families of the taxa.

The COI dataset of 263 North American bird species [23] included 437 DNA sequences from 157 genera, 50 families and 20 orders (Table 1). In the K2P/NJ tree (Figures 1 and 2), species from 18 orders could be properly grouped, except that *Falco peregrinus, F. columbarius, F. sparverius *and *Cathartes aura *from the order Falconiformes and *Grus canadensis *from the order Gruiformes did not cluster with members of the same order. At the family level, species from 45 families were grouped respectively. All species could be correctly grouped and identified at the genus/species level (Table 2). In the CV/NJ tree (Figure 3), other than species from the above two orders, members of two other orders, Passeriformes and Ciconiiformes, could not be grouped together. In the former, two taxa (of 180) were clustered with members in the order Columbiformes, while one species (of four) from Ciconiiformes was nested within the Gruiformes species. At the family level, species from 43 families could be grouped correctly in our CV/NJ tree (Figure 4). Compared with the K2P/NJ tree, species from two additional families failed to cluster together. One species (out of 11), *Oenanthe oenanthe*, from the family Turdidae and three species (out of 13), *Melospiza georgiana, M. lincolnii *and *M. melodia*, from the Emberizidae could not be assigned to their respective families. As in the K2P/NJ method, all species could be grouped and identified at the genus/species level in the CV/NJ tree (Table 2). The CV method matched the overall grouping effectiveness with the K2P analysis in this dataset.

**Table 1:** Details of datasets and *K *values used in CV analysis

**Taxa**	**Gene**	**Avg. sequence length (bp)**	**No. of classes**	**No. of orders**	**No. of families**	**No. of genera**	**No. of species**	**No. of sequences**	***K***
Birds	COI	667	1	20	50	157	263	437	9
Fishes	COI	649	4	14	49	113	207	754	9
Nematodes	SSU	1,693	12 clades					349	10
Decapods	16S	450	1	1	86	323	734	734	9

**Table 2:** Grouping and processing time using the K2P (with CLUSTAL W alignment) and CV methods

**Taxa**		**Birds**			**Fishes**			**Nematodes**	**Decapods**		
		**orders**	**families**	**genera**	**orders**	**families**	**genera**	**clades**	**Infraorders**	**families**	**genera**
Grouping	K2P	18/20	45/50	All	10/14	45/49	All	12/12	3/8	80/86	All
	CV	16/20	43/50	All	10/14	39/49	All	9/12	1/8	79/86	All
Processing time	K2P		>5 h			>10 h		> 10 h		> 10 h	
	CV		<3 min			< 5 min		< 5 min		< 5 min	

### Fish dataset

**Figure 5:**
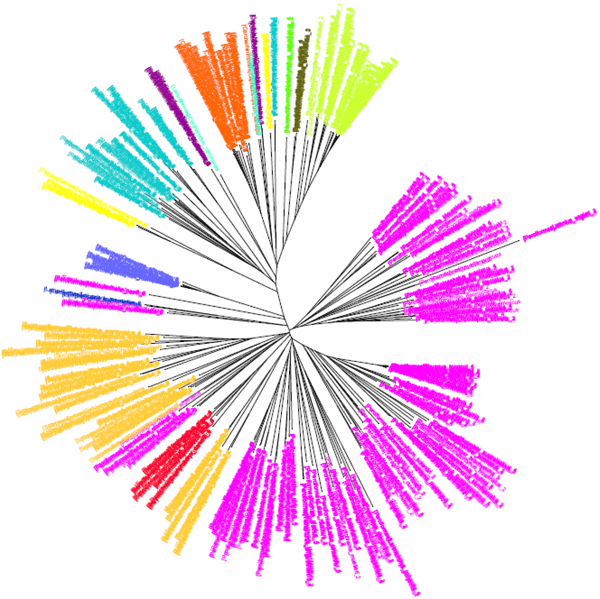
**K2P/NJ tree based on COI dataset of fishes (Ward et al 2005).**Sequences are labelled in different colours according to the orders of the taxa.

**Figure 6:**
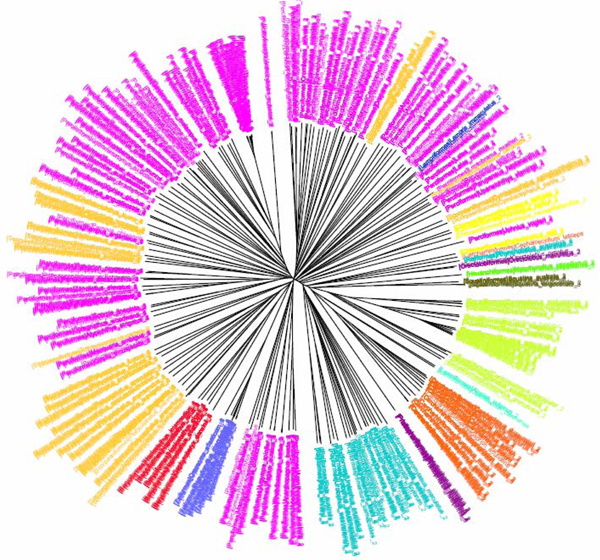
**CV tree (*K*=9) based on COI dataset of fishes (Ward et al 2005).**Sequences are labelled in different colours according to the orders of the taxa.

**Figure 7:**
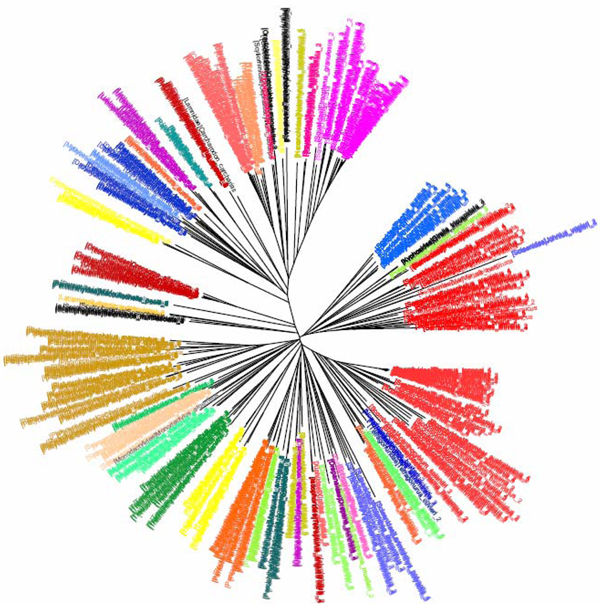
**K2P/NJ tree based on COI dataset of fishes (ward et al 2005).**Sequences are labelled in different colours according to the families of the taxa.

**Figure 8:**
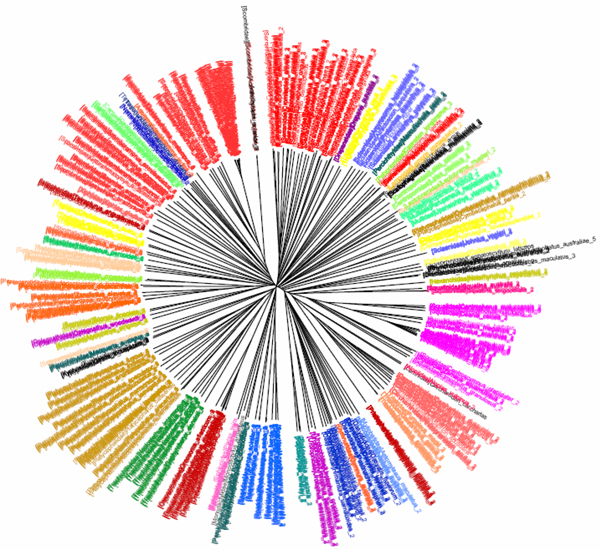
**CV tree (*K*=9) based on COI dataset of fishes (Ward et al 2005).**Sequences are labelled in different colours according to the families of the taxa.

The COI dataset of 207 Australian marine fish species [5] contained 754 COI sequences with an average length of 649 bp from 4 classes, 14 orders, 49 families and 113 genera (Table 1). In the K2P/NJ tree (Figure 5), 42, 5, 1 and 1 species of the orders Perciformes (total 96 species), Scorpaeniformes '(25 species)' Rajiformes (21 species) and Lamniformes (2 species) could not be grouped with members of the same order, respectively. These former orders also failed to be grouped accordingly in the CV/NJ tree (Figure 6) but all species from the order Lamniformes could be successfully grouped together. Yet the CV/NJ tree failed to assign *Callorhinchus milii*, a species of Chimaeriformes (total 4 species included), to the clade of this order. At the family level (Figure 7), four out of the 49 families could not be grouped correctly in the K2P/NJ tree. 4, 1, 1 and 1 species of the families Dasyatididae (total 10 species), Percichthyidae (4 species), Sciaenidae (6 species) and Scorpidae (3 species) could not be grouped with members from the same family, respectively. In the CV/NJ tree (Figure 8), members from six additional families failed to be grouped properly. 1, 2, 3, 8, 2 and 2 species of the families Myliobatidae (total 3 species), Platycephalidae (14 species), Serranidae (24 species), Scombridae (24 species), Scorpaenidae (5 species) and Triglidae (5 species) could not be grouped with members from the same family. As with the K2P/NJ tree, all 207 species could be correctly discriminated and identified at the genus/species level with the CV/NJ method (Table 2). The CV method matched the resolving power of the K2P method in terms of its effectiveness in identifying species.

### Nematode dataset

**Figure 9:**
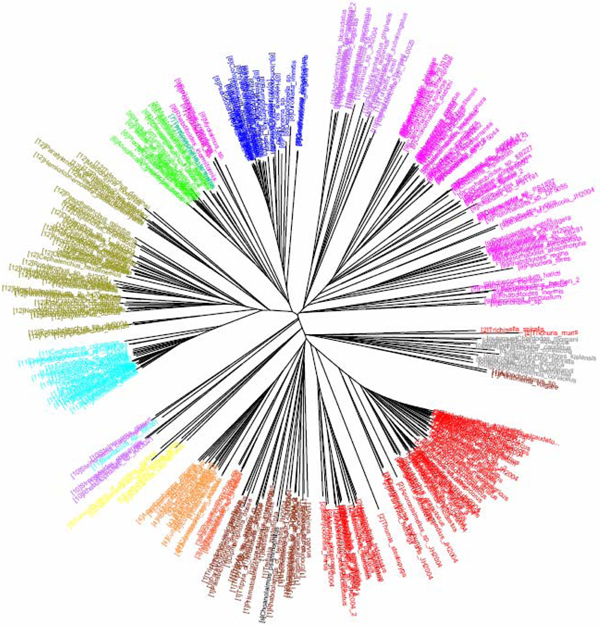
**CV tree (*K*=10) based on SSURNA dataset of nematodes (Holterman et al 2006).**Sequences are labelled in different colours according to the 12 clades defined by Holterman et al (2006).

Holterman et al [14] used a small-subunit (SSU) rDNA tree to reconstruct the phylogenetic relationships in the phylum Nematoda. The dataset of nearly full-length SSU rDNA sequences (average length 1,693 bp) contained 339 ingroup taxa and ten outgroup taxa (Table 1). Based on three phylogenetic reconstruction methods, including Bayesian inference (BI), maximum parsimony (MP) and NJ, the species could be separated into 12 major clades. The CV/NJ tree (Figure 9) was also able to generate a similar tree topology with the 12 clades, and successfully assigned 326 (of 339) taxa into their respective clades (Table 2), except two species in clade 1 (*Adoncholaimus *sp. and *Pontonema vulgare*), two species from clade 2 (*Trichinella spiralis *and *Trichuris muris*), four species from clade 9 (*Aduncospiculum halicti*, *Pristionchus lheritieri*, *Pristionchus pacificus *and *Rhabditoides inermis*), five species from clade 10 (*Steinernema carpocapsae, Steinernema glaseri*, *Rhabditophanes *sp., *Strongloides ratti *and *Strongyloides stercoralis*) and one species from clade 11 (*Brevibucca *sp.). The four species from clades 1 and 2 were placed in the basal position in the CV/NJ tree while the five species from clade 9 formed a sister group to the group comprising clades 6 and 7. The five species in clade 10 were clustered with *Brevibucca *sp. from clade 11. Holterman et al [14], using traditional methods, were not able to suggest a phylogenetic position of the family Choanolaimidae. With the CV method, changing the *K *value would alter the position of Choanolaimidae. For instance, with the *K *value of 10 Choanolaimidae was assigned into clade 1. When the *K *value dropped below 10, Choanolaimidae became a sister group to the group comprising clades 3 and 4.

### Decapod dataset

**Figure 10:**
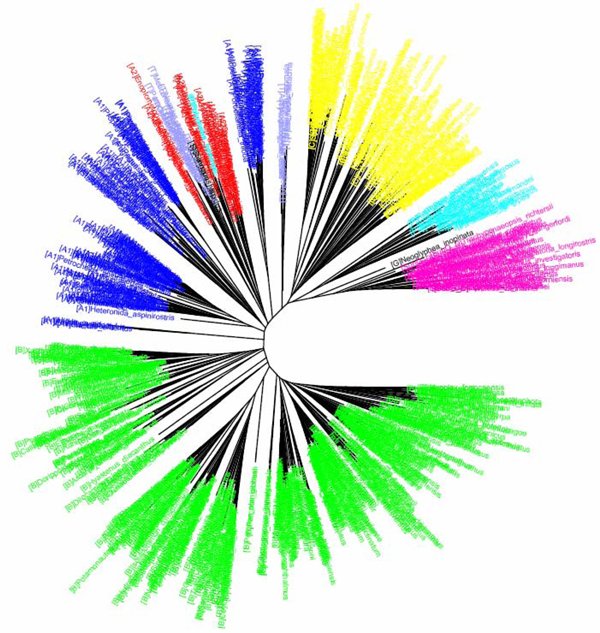
**K2P/NJ tree based on the 16S rRNA dataset of decapod crustaceans.**Sequences are labelled in different colours according to the infraorders of the taxa.

**Figure 11:**
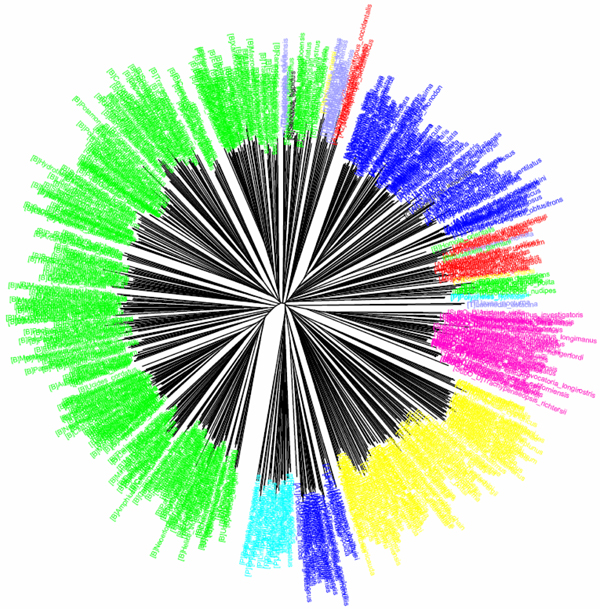
**CV tree (*K*=9) based on the 16S rRNA dataset of decapod crustaceans.**Sequences are labelled in different colours according to the infraorders of the taxa.

The 16S rRNA dataset of decapod crustaceans contained 734 sequences (each from one species) from eight infraorders, 42 superfamilies, 86 families and 323 genera (Table 1). The average sequence length was 450 bp. Both the K2P/NJ and CV/NJ trees showed similar topologies, and the relationships among the taxa were also highly similar between the two trees. At the infraorder level, neither tree could group the 134 Anomura species together. In the K2P/NJ tree (Figure 10), species from the infraorder Anomura were separated into three major groups, while only two were evident in the CV/NJ tree (*K *= 9). For the infraorder Astacidea, the CV/NJ tree (Figure 11) separated the 27 species into two clades. Although the K2P/NJ tree was able to group all these species into a single clade, the clade also contained species from two other infraorders, Thalassinidea and Palinura. Neither tree was able to assign infraorders Thalassinidea (15 species) and Palinura (28 species) as reciprocal monophyletic groups. By contrast, all the 375 species from the infraorder Brachyura could be successfully grouped in the K2P/NJ tree, while 11 of the species could not be grouped with other members of this infraorder by the CV/NJ method. In fact, the Brachyura clade in the CV/NJ tree also contained a small number of species from the infraorder Thalassinidea. By using the K2P/NJ method, all 108 species from infraorder Caridea could be successfully grouped as a single clade, while three species could not be grouped into the Caridea clade in the CV/NJ tree. At the family level, both the K2P and CV methods showed a high resolving power in assigning taxa to their respective families. With the K2P/NJ method, only members from six families, viz. Ocypodidae, Oplophoridae, Palaemonidae, Palinuridae, Penaeidae and Potamonautidae, could not be grouped respectively, out of a total of 86 families. Interestingly, while species from Penaeidae could be grouped together in the CV/NJ tree, members from two other families, Atyidae and Gecarcinidae, failed to cluster together in this tree. The overall grouping effectiveness of the K2P and CV methods was comparable (Table 2).

### Processing time

All the data analyses in this study were preformed on a 1.4 GHz notebook computer. With the K2P/NJ method, the alignment step using CLUSTAL W [24] alone took more than five hours to complete for the bird dataset, and required more than 10 hours to complete for the other three datasets (Table 2). When the other two faster algorithms MAFFT [25] and MUSCLE [26] were used, the processing time was reduced by 3.2 and 7.7 folds respectively, so that between one and two hours were needed for aligning the bird dataset. By contrast, with the CV method it took less than three minutes to analyze this bird dataset, and the analysis of the other three datasets was completed in five minutes (Table 2). Thus, while the CV/NJ method matched the resolving power of the K2P/NJ method in generating a highly similar tree, it took less than 6% of the time needed in sequence alignment on the same computer.

## Discussion

Qi et al [22] first introduced the CV method to analyze the phylogeny of prokaryotes based on the complete genomes, and this approach has subsequently been applied to analyze the chloroplast genomes [13,27]. All these studies were based on protein sequences, and a procedure to subtract the random background from the frequencies of oligopeptide strings was used before computation of the CVs in order to diminish the influence of random neutral mutations at the molecular level and to highlight the shaping role of selective evolution. However, in adopting the CV method in analyzing short rDNA sequences for barcoding, Chu et al [13] have shown that a subtraction procedure for random background does not further enhance the reliability of the groupings. In the present study, we focus on how well the CV method could handle large DNA datasets, by comparing this method with the K2P/NJ method in analyzing four datasets including both rRNA and protein-coding genes. Although in each of the four datasets tested the topologies between the CV/NJ and K2P/NJ trees were not identical, this does not mean that one method obtained better results than the other. The rationale for generating the K2P/NJ trees in DNA barcoding studies or in BOLD is that the K2P/NJ tree construction is relatively simple [1], so that a query sequence can be rapidly identified to the species level. However, the K2P/NJ tree was not intended to reflect the phylogenetic relationships among the taxa analyzed [5]. Thus any detailed comparison in tree topologies between the CV and the K2P methods is meaningless. In fact, both the CV and K2P methods performed equally well in identifying and discriminating taxa at the genus/species level in the datasets tested (Table 2).

The dataset of nematodes [14] was used to resolve the phylogenetic relationships among the species, and three different approaches (BI, MP and NJ) were applied. However, the classification of nematodes based on the molecular approach is never an easy task. The phylogenetic position of the family Choanolaimidae, for example, could not be determined based on any of the three approaches. Two species, *Trichinella spiralis *and *Trichuris muris*, could only be assigned into clade 2 based on the BI tree but not on the MP and NJ trees [14]. The problem might be due to the inadequacy of the SSU rDNA sequences in resolving the phylogeny of this group. The evolutionary rates of this gene may also differ among different nematode clades [14]. On the other hand, Floyd et al [15] proposed to use the number of SSU rDNA sequence differences to define "molecular operational taxonomic units" rather than adopting the classical species concepts in nematode biodiversity studies. It is not surprising to find inconsistent tree topologies based on different analytical methods, including the CV method, from this nematode dataset. In fact, the CV method could generate a tree topology similar to those generated by the three phylogenetic reconstruction methods, by clustering all of the taxa into 12 main clades (Table 2).

Besides its impressive resolving power, the main advantage of the CV/NJ method over the K2P/NJ algorithm is its speed. In every dataset analyzed in this study, the CV/NJ method could generate trees in less than five minutes on a 1.4 GHz notebook computer. The time needed is at least 15-fold more using the K2P/NJ method, which requires sequence alignment. Given its high analytical speed, the CV method could profitably serve as a quick barcoding identification tool capable of matching a query sequence against a pre-installed reference dataset from BOLD on a notebook computer for field workers who may not have internet access. The CV method not only saves time by omitting the alignment step, but also avoids the introduction of any human errors during the alignment process. Where no universal alignment parameters can be defined, every gap representing insertion/deletion that is assigned to a DNA sequence should be checked by eye carefully, and even this step might be subjective [19]. Manual checking becomes a very tedious and laborious procedure when dealing with a large dataset generated by multiple alignments, but is a necessary step for verifying the reliability of the dataset and its suitability for further analysis. The CV method does away with this rate-limiting, tedious step in the tree construction procedure for DNA barcoding.

## Conclusion

The CV method was first demonstrated to facilitate the use of rDNA datasets for barcoding purposes since no sequence alignment was necessary [13]. In the present study, we further demonstrated the power of the CV method in analyzing large DNA barcode datasets, regardless of the type of gene markers used. In all cases tested, the CV/NJ method achieved tree topologies similar to those based on the traditional methods which involve sequence alignment, with compatible grouping effectiveness. Furthermore, when the CV method was used, the computational time was at least 15-fold shorter than that based on the K2P method. Besides its effective resolving power and very fast speed of analysis, the CV/NJ method can routinely generate reliable and reproducible trees by eliminating human errors in the multiple alignment process. To conclude, we propose that the CV/NJ method can be used as an effective and efficient tree construction algorithm in analyzing DNA barcode datasets.

## Methods

Sequences from three published datasets, including birds [23], fishes [5] and nematodes [14] were downloaded from GenBank for analysis. We also assembled a 16S rRNA dataset of decapod crustaceans by including 466 sequences from GenBank and 268 sequences generated in our laboratory. This dataset is available from the first author upon request.

The principle and details of the composition vector (CV) method have been fully described previously [13,22,28,29], and the program is publicly available at . In short, for a sequence of gene of length *L*, the frequency of the appearance of oligonucleotide strings of a fixed length *K *was determined. The *K *value used for each dataset was calculated by [30] and it ranged from 9 to 10 among the four datasets (Table 2). The total number of *N *possible types of the *K *strings was 4^*K*^. The frequency of each of the *N *kinds in a given DNA sequence was determined. We then placed the frequencies of all possible *K*-strings in a fixed order to obtain a CV of dimension 4^*K *^for each sequence. The correlation *C(A, B) *between two sequences *A *and *B *was determined by taking the projection of one vector on another, and the distance between the two was defined as *D *= (1 - *C*)/2. In this way, the sequences difference could be quantitatively evaluated. After constructing a distance matrix for all sequences in a dataset, the neighbour-joining (NJ) [31] analysis implemented in Phylip 3.63 [32] was used to construct a profile tree for each dataset. The CV/NJ trees were then compared with the corresponding K2P/NJ trees constructed as follows. The DNA sequences in each dataset were first aligned using the multiple alignment program CLUSTAL W 1.5c [24], and the NJ trees were constructed using Mega 3 [33] based on the Kimura-2-parameter distance model [34]. We also attempted to align the sequences using two other algorithms, MAFFT [25] and MUSCLE [26]. All the computation was performed using a 1.4 GHz notebook computer with 512 MB of RAM.

## Competing interests

The authors declare that they have no competing interests.

## Authors' contributions

K. H. Chu designed the study and wrote the final draft of the manuscript. M. Xu collated the sequences and conducted the analysis. C.P.. Li assisted in data analysis and manuscript preparation.
